# Prevalence of Exclusive Breastfeeding in Bangladesh and Its Association with Diarrhoea and Acute Respiratory Infection: Results of the Multiple Indicator Cluster Survey 2003

**Published:** 2007-06

**Authors:** Seema Mihrshahi, Naomi Ichikawa, Muhammad Shuaib, Wendy Oddy, Rose Ampon, Michael J. Dibley, A.K.M. Iqbal Kabir, Jennifer K. Peat

**Affiliations:** 1 Children's Hospital at Westmead, Sydney, NSW, Australia; 2 Curtin University of Technology, Perth, WA, Australia; 3 ICDDR,B, GPO Box 128, Dhaka 1000, Bangladesh; 4 Office of Emergency Programmes, UnitedNations Children's Fund, New York, USA (formerly with Planning, Monitoring and Evaluation Section, United Nations Children's Fund, Dhaka, Bangladesh); 5 Institute of Statistical Research and Training, University of Dhaka, Dhaka, Bangladesh; 6 Telethon Institute of Child Health, Perth, WA; 7 University of Newcastle, Newcastle, NSW, Australia

**Keywords:** Acute respiratory infections, Breastfeeding, Cluster surveys, Cross-sectional studies, Diarrhoea, Diarrhoea, Infantile, Infant-feeding practices, Bangladesh

## Abstract

The objective of this study was to investigate the association between the prevalence of exclusive breastfeeding and morbidity (diarrhoeal diseases and acute respiratory infection) in infants aged 0-3 month(s) using the Multiple Indicator Cluster Survey (MICS) 2003 data from Bangladesh. The study population included 1,633 infants aged 0-3 month(s). The prevalence of diarrhoea and acute respiratory infection was compared using the chi-square tests between infants aged 0-3 month(s) who were exclusively breastfed and infants who were not exclusively breastfed. Logistic regression was used to adjust for confounders and for calculating adjusted odds ratios. To adjust for cluster sampling and reduced variability, the adjusted chi-square value was divided by the design effect, and a re-estimated p value was calculated. The prevalence of diarrhoea and acute respiratory infection in this sample of 0-3-month old infants in Bangladesh was 14.3% and 31.2% respectively. The prevalence of both illnesses was significantly associated with lack of exclusive breastfeeding. The adjusted odds ratio for diarrhoea was 0.69 (95% confidence interval [CI] 0.49-0.98, p=0.039), and the adjusted odds ratio for acute respiratory infection was also 0.69 (95% CI 0.54-0.88, p=0.003). Only 192 infants (11.7% of total sample) were exclusively breastfed at the time of interview, and 823 infants (50.3%) were never exclusively breastfed. The prevalence of prelacteal feeding was 66.6%. The results confirmed a protective effect of exclusive breastfeeding against infectious diseases-related morbidity in infancy and showed that frequently-collected cross-sectional datasets could be used for estimating effects. The low prevalence of exclusive breastfeeding in Bangladesh needs to be improved to decrease child morbidity.

## INTRODUCTION

In Bangladesh, infectious diseases, such as diarrhoea and acute respiratory infections, are a cause of more than two-thirds of all deaths in children aged less than one year (1). The importance of breastfeeding in the prevention of infectious diseases during infancy is well-documented (2–7). Breastmilk provides protection against pathogens by providing antibacterial and antiviral substances that stimulate the infant's immune system (6). A meta-analysis of data from three developing countries showed that infants who were not breastfed had a six-fold greater risk of dying from infectious diseases in the first two months of life than those who were breastfed (3), and a similar protective effect of breastfeeding has been shown in studies of morbidity from infectious diseases (8–10). A recent estimate from the Bellagio Child Survival Study Group, which used results of systematic reviews from low- and middle-income countries, predicted that exclusive breastfeeding in the first six months of life and continued breastfeeding for the first year could prevent 1.3 million child deaths worldwide, making promotion of breastfeeding a key strategy of child-survival programmes (11).

Exclusive breastfeeding means that the infant receives no solids or liquids apart from breastmilk with the exception of vitamins, minerals, or medicines (12). Infants who are exclusively breastfed are less likely to be exposed to contaminated foods and liquids, and this contributes to reductions in the incidence and severity of infectious diseases. Currently, the recommendation from the Global Strategy for Infant and Young Child Feeding, developed by World Health Organization (WHO) and United Nations Children's Fund (UNICEF), is that infants should be exclusively breastfed for the first six months of life (13). After six months, infants should receive nutritionally-adequate and safe complementary foods while continuing to be breastfed for up to two years of age or beyond. Recent estimates predict that current breastfeeding patterns are far below the recommended levels especially in Africa and Asia where rates of exclusive breastfeeding for the first six months are less than 40% (14).

Factors that interact with the protective effect of breastfeeding include environmental, cultural and economic characteristics. The protective effect of breastfeeding is most important in populations with high infant mortality, high illiteracy, poor sanitation facilities, poor nutritional status, and generally low economic status (6). The population of Bangladesh fits all of these criteria (15).

Surveys of child-feeding practices from Bangladesh showed an almost universal continuation of any breastfeeding up to two years of age (15). However, cultural practices include the feeding of prelacteal foods, such as honey, sugar water, or mustard oil immediately after birth contributing to the low prevalence of exclusive breastfeeding (16). Current data show that 38% of children aged 2-3 months are exclusively breastfed, and 23% of children are given complementary foods before the sixth month (15). In addition, rates of bottlefeeding are high with 30% of infants aged 2-3 months being bottlefed. The rate of consumption of baby formula in infants aged 4-7 months has almost doubled since 2000 and is highest in urban areas (15).

This paper describes the infant-feeding practices in Bangladesh using the Multiple Indicator Cluster Survey (MICS) 2003 data and investigates the association between exclusive breastfeeding and child morbidity, in particular diarrhoea and acute respiratory infection.

## MATERIALS AND METHODS

### Data analysis

Cross-sectional data from the Multiple Indicator Cluster Survey (MICS) from Bangladesh collected in 2003 were used for analyzing the association between breastfeeding and diarrhoea and acute respiratory infection in infants aged 0-3 month(s).

### Data of multiple indicator cluster surveys

MICS are nationally representative surveys of households, women, and children and commonly include over 5,000 households. The surveys include information about the duration and patterns of breastfeeding and complementary feeding practices, childhood illnesses, education, vaccination coverage, and sanitation. Bangladesh Bureau of Statistics, under the Monitoring the Situation of Children and Women Project, supported by UNICEF, collected data for the MICS 2003 in Bangladesh from 63,420 households. UNICEF Bangladesh provided the datasets.

### Prevalence of exclusive breastfeeding

The MICS 2003 provides two types of data for calculating the prevalence of exclusive breastfeeding—one allowing for prelacteal feeding and the other not allowing for prelacteal feeding, the ‘true’ rate of exclusive breastfeeding. This classification is to enable valid comparisons with other surveys, such as the demographic and health surveys (DHSs) which allow for prelacteal feeding in their calculation of rate of exclusive breastfeeding. For the purposes of this analysis, the definition of exclusive breastfeeding included those children who had been given prelacteal foods.

Table 1 shows the questions relating to the outcomes (morbidity) and study factors (breastfeeding); all these questions were taken from Part B of the survey, the questionnaire relating to children aged less than five years.

**Table 1:** Questions relating to explanatory variables and outcomes

Variable	Question (s)
Breastfeeding status	
Prelacteal feeding	Q12: (For children aged 0-23 month(s)) Did you give honey/sugar water/mustard oil/other to your child immediately after birth?
Initiation of breastfeeding	Q12a: (For children aged 0-23 month(s)) Was your child given breastmilk within three days of birth ?
Duration of breastfeeding	Q13: How long had the child been breastfed? (months)
Duration of exclusive breastfeeding outcomes	Q13a: (For children aged 0-23 month(s)) How long was the child exclusively breastfed? (months)
Prevalence of diarrhoea	Q5: Did the child have loose watery motions three or more times in a day during the last two weeks?
Prevalence of acute respiratory infection	Q10: Did your child have ‘cough and/or difficulty breathing' in the last two weeks?
	Q11: If yes, what was happening to the child? Multiple responses with the following symptoms: simple cough, runny nose, fever, fast breathing, chest indrawing, inability to eat/ drink, or convulsions, excessive sleepiness

A derived variable—Probable acute respiratory infection—was defined as a positive response to Q10 and a positive response to one or more of the following symptoms as assessed by Q11: fever, fast breathing, chest indrawing, inability to eat/drink or convulsions or excessive sleepiness.

### Statistical analysis

Data were analyzed using the SPSS software (version 13.0) (SPSS Inc., Chicago, IL). The prevalence of diarrhoea and acute respiratory infection in the last two weeks was compared using the chi-square tests between children aged 0-3 month(s) who were exclusively breastfed and children who were not exclusively breastfed. Univariate odds ratios (95% confidence interval [CI]) were calculated. Logistic regression was used for adjusting for gender and age of child, number of siblings in household, stratum, ownership of household, source of drinking-water, place of disposal of faeces, and education of mother, and multivariate odds ratios are also reported.

To adjust for cluster sampling and reduced variability in the sample, a one-way ANOVA was used for calculating the intraclass correlation coefficient (ICC). This was then used in the following equation for the design effect to be calculated.

Design effect=1 + (m-1) ∗ ICC where m=average cluster size

A re-estimated chi-square value was then calculated by dividing it by the design effect. A re-estimated p value was also calculated.

### Ethical approval

Ethical approval for this analysis was obtained from Human Research Ethics Committees of Curtin University of Technology, Perth, Western Australia and ICDDR,B, Dhaka, Bangladesh.

## RESULTS

In total, 1,633 children aged three months or under had data available for analysis during the MICS 2003 period between 7 March 2003 and 23 September 2003. Characteristics of the population are described in Table 2. Most (n=1,515, or 92.8%) of the children were not registered at birth by a local authority.

**Table 2:** Demographic characteristics of children/families (n=l,633) surveyed

Characteristics	No.	Percentage
Gender of child		
Male	790	48.4
Female	843	51.6
Age (months) of child		
0	334	20.5
1	368	22.5
2	396	24.2
3	535	32.8
Birth registered		
Yes	118	7.2
No	1,515	92.8
Stratum		
Rural	1,128	69.1
Metro-city—non-slum	118	7.2
Metro-city—slum	55	3.4
District—urban	276	16.9
Tribal	56	3.4
Education of mothers∗		
Illiterate	670	42.0
Primary	410	25.7
Secondary	485	30.4
Higher	29	1.8
Education of household heads		
Illiterate	859	52.6
Primary	348	21.3
Secondary	357	21.9
Higher	69	4.2
Ownership of household		
Own house	1,372	84.0
Rent	261	16.0
Source of drinking-water		
Tubewell/tap/ringwell	1,549	94.9
Pond/river/other	75	4.5
Both	10	0.6
Place of disposal of faeces		
Latrine/hole/fixed place	1,210	74.1
No fixed place	423	25.9

∗n=l,594 as in 39 cases the mother was not the primary carer of the child

The prevalence of breastfeeding practices among the families surveyed is shown in Table 3. More than 99% of the children were still breastfeeding at the time of interview, but only 34.5% were being exclusively breastfed (prelacteal feeding included). If the WHO definition of exclusive breastfeeding is used, only 192 (11.7%) children were exclusively breastfed at the time of interview, In total, 823 (50.3%) infants were never exclusively breastfed, 1,450 (88.8%) infants were given breastmilk within three days of birth, and 66.6% of infants were given a prelacteal feed of honey/sugar water or mustard oil after birth.

**Table 3:** Prevalence of breastfeeding practices in 1,633 infants

Breastfeeding practice	No.	Percentage
Any breastfeeding	1,620	99.2
Exclusive breastfeeding (with prelacteal feed)	564	34.5
Exclusive breastfeeding (WHO definition)	192	11.7
Prelacteal feed given	1,088	66.6
Breastmilk given within 3 days of birth	1,450	88.8

WHO=World Health Organization

Table 4 shows the prevalence of exclusive breastfeeding and illness by age of the child. The prevalence of exclusive breastfeeding in infants aged three months was less than 20%. The prevalence of diarrhoea and acute respiratory infection was highest in infants aged three months and followed a linear trend with increasing age.

**Table 4:** Prevalence of exclusive breastfeeding and illness by age of child

Age (months)	Exclusive breastfeeding		Prevalence of diarrhoea		Exclusively breastfed infants with diarrhoea		Prevalence of acute respiratory infection		Exclusively breastfed infants with ARI	
	No.	%	No.	%	No.	%	No.	%	No.	%
0	184	55.1	15	4.5	8	4.3	56	16.8	28	15.2
1	159	43.2	36	9.8	13	8.2	103	28.0	46	28.9
2	117	29.5	73	18.4	13	11.1	131	33.1	21	17.1
3	104	19.4	110	20.6	21	20.2	219	40.9	35	33.7

ARI=Acute respiratory infection

The relationship between exclusive breastfeeding and the prevalence of diarrhoea and acute respiratory infection is shown in Table 5. In total, 14.3% of the children had diarrhoea in the last 15 days. Over half (53.6%) of the children in the survey had cough or difficulty breathing in the last 15 days, and 509 (31.2%) children had probable acute respiratory infection.

**Table 5:** Unadjusted and adjusted odds ratios for diarrhoea and acute respiratory infection in the last 2 weeks in infants exclusively breastfed at 0-3 month(s) compared with infants who were not exclusively breastfed (n=1,633)

Disease	No. with symptoms	Percentage	Prevalence in the last 2 weeks		Percentage of difference	Odds ratio (95% CI)	p value	Adjusted odds ratio∗ (95% CI)	p value†
			Non-exclusively breastfed group %	Exclusively breastfed group %					
Diarrhoea	234	14.3	16.7	9.8	6.9	0.54 (0.39-0.74)	<0.0001	0.69 (0.49-0.98)	0.038
Acute respiratory infection	509	31.2	35.5	23.0	12.5	0.55 (0.43-0.69)	<0.0001	0.69 (0.54-0.88)	0.003

∗Adjusted for gender and age of child, number of siblings in household, stratum, ownership of household, source of drinking-water, place of disposal of faeces, and education of mothers; ^†^p value adjusted for cluster sampling CI=Confdence interval

The prevalence of diarrhoea in this population was significantly associated with lack of exclusive breastfeeding. The unadjusted odds ratio was 0.54 (95% CI 0.39-0.74, p<0.0001). After adjustment for a number of factors, including gender and age of child, number of siblings in the household, stratum, ownership of household, source of drinking-water, place of disposal of faeces, and education of mother, the adjusted odds ratio was 0.69 (95% CI 0.49-0.98, p=0.039). Table 6 shows the odds ratios for the variables in the model. Apart from exclusive breastfeeding, the only other significant predictors of diarrhoea were age of the infant and source of drinking-water for the family.

**Table 6:** Variables included in the logistic regression model for the effect of exclusive breastfeeding on diarrhoea and acute respiratory infection

Variable	Odds ratio for diarrhoea (95% CI)	Odds ratio for ARI (95% CI)
Exclusively breastfed		
Yes	0.69 (0.49-0.98)∗	0.69 (0.54-0.88)∗
No	1.0	1.0
Age (months) of child Continuous scale	0.62 (0.54-0.72)∗	0.72 (0.65-0.80)∗
Gender of child		
Male	1.09 (0.82-1.45)	0.92 (0.74-1.15)
Female	1.0	1.0
Education of mothers		
Illiterate	1.0	1.0
Primary	1.38 (0.95-2.00)	1.52 (1.15-2.00)∗
Secondary or higher	0.88 (0.62-1.24)	1.14 (0.86-1.49)
Number of siblings		
0	1.10 (0.79-1.55)	0.92 (0.71-1.19)
1	1.30 (0.87-1.93)	0.95 (0.71-1.27)
≥2	1.0	1.0
Stratum		
Tribal	1.0	1.0
Rural	1.21 (0.52-2.82)	0.42 (0.19-0.92)∗
Metro-city—non-slum	1.88 (0.65-5.44)	0.83 (0.33-2.08)
Metro-city—slum	1.27 (0.39-4.11)	0.47 (0.17-1.28)
Divisional other urban	1.41 (0.56-3.52)	0.46 (0.20-1.04)
Ownership of household		
Own home	1.36 (0.85-2.15)	1.24 (0.87-1.78)
Rented	1.0	1.0
Source of drinking-water		
Tap/tubewell/ringwell	0.42 (0.17-0.99)∗	0.62 (0.35-1.07)
Pond/river/other	1.0	1.0
Disposal of faeces		
Fixed place	0.86 (0.60-1.23)	0.98 (0.75-1.27)
No fixed place	1.0	1.0

∗p<0.05, significant variables in the model ARI=Acute respiratory infection; CI=Confidence interval

The prevalence of acute respiratory infection was also significantly associated with lack of exclusive breastfeeding. The unadjusted odds ratio for acute respiratory infection was 0.55 (95% CI 0.43-0.69). After adjustment the above factors, the adjusted odds ratio was 0.69 (95% CI 0.54-0.88, p=0.003). Table 6 shows the odds ratios for the variables in the model. Other significant predictors of acute respiratory infection apart from exclusive breastfeeding were age of the infant, stratum (rural), and education of mother.

## DISCUSSION

These results confirm a protective effect of exclusive breastfeeding against infectious diseases-related morbidity in infancy. The observed protection remained even after adjustment for a number of confounders, including demographic variables (age and gender of child), socioeconomic variables (education of mother, stratum, ownership of household), and sanitation variables (source of drinking-water and place of disposal of faeces). The results showed that children, aged 0-3 month(s), who are exclusively breastfed were less likely to have suffered from diarrhoea (adjusted OR=0.69, 95% CI [0.49-0.98]) or an acute respiratory infection [adjusted OR=0.69, (95% CI 0.54-0.88)] than infants who were not exclusively breastfed. The results are consistent with those of other studies on the association between mode of feeding and morbidity in children (8–10).

In peri-urban Mexico City, a home-based peer-counselling intervention was conducted to increase rates of exclusive breastfeeding (8). At three months of age, the proportion of infants exclusively breastfed in intervention groups was more than double the proportion in control groups, and this resulted in a two-fold decrease in diarrhoeal illness (26% vs 12%, p=0.029). This was comparable with the MICS 2003 analysis where the prevalence of diarrhoeal illness in the exclusively breastfed group was reduced by almost half (16.7% vs 9.8%, p<0.001).

A larger-scale randomized trial—The Promotion of Breastfeeding Intervention Trial (PROBIT)—was a multicentre study conducted in the Republic of Belarus in which the maternity-care centres were allocated to an exclusive breastfeeding intervention or control (9). In total, 17,046 mother-infant pairs were enrolled, and 16,491 (96.7%) were followed up to the age of one year. Infants from intervention sites were more likely to be exclusively breastfed at three months (43.3% vs 6.4%; p<0.001). In addition, infants from intervention sites had a significant reduction in the risk of gastrointestinal tract infections in the first year (13.2% vs 9.1%; adjusted OR=0.60 [95% CI, 0.40-0.91]), but no significant reduction in respiratory tract infection (39.2% vs 39.4%; adjusted OR=0.87 [95% CI 0.59-1.28]) was observed. The MICS analysis showed a similar OR for diarrhoeal illness, adjusted OR=0.69 (95% CI, 0.49-0.98). We, however, also showed a significant reduction in respiratory tract illness due to exclusive breastfeeding (adjusted OR 0.69 [95% CI 0.54-0.88]). A possible explanation for this is the difference in the socioeconomic status between the populations in Belarus and Bangladesh; thus, the possibility of showing an effect in Bangladesh would be greater.

In a cluster-randomized controlled trial in India, eight communities were randomized to receive an educational counselling intervention to promote exclusive breastfeeding for six months or to a control with no specific intervention (10). In total, 1,115 mother-infant pairs were enrolled, and 880 (79%) remained in the study at six months. At three months, rates of exclusive breastfeeding were 79% in intervention communities and 48% in control communities (p<0.0001). The seven-day prevalence of diarrhoea was significantly lower in intervention than in control communities at three months (OR=0.64 [95% CI 0.44-0.95], p=0.028), and these results are similar to the MICS 2003 analysis.

Although the prevalence of any breastfeeding in Bangladesh is high, the major barriers to achieving the recommendations of the Global Strategy for Infant and Young Child Feeding include a low prevalence of exclusive breastfeeding and a high prevalence of prelacteal feeding.

The MICS 2003 data showed that, in a representative sample of infants aged 0-3 month(s), only 34.5% were being exclusively breastfed. If the WHO definition of exclusive breastfeeding was used, only 11.7% were being exclusively breastfed. These rates are comparable with recently-published rates from the DHS 2004 which showed that 38% of infants aged 2-3 months were being breastfed exclusively (15). Results of an earlier study in 1992 showed that the prevalence of exclusive breastfeeding was 20% at five months, and 16% of infants had been given bottle-feeds by the age of one month (17). A limitation in the MICS 2003 dataset is that the prevalence of feeding other liquids, such as juices and formula and solids, are not available and, therefore, the prevalence predominant or partial breastfeeding was not calculated. In another study in 1996, only 15% of infants were exclusively breastfed to five months of age (18). These results showed that the prevalence of exclusive breastfeeding has not improved, and only 11.7% of children aged 0-3 month(s) are being fed according to the recommended guidelines.

The demographic, health and social factors can affect the prevalence of exclusive breastfeeding. In one study, mothers who were more educated were less likely to breastfeed their children exclusively for the first five months. The authors suggest that education is a proxy for socioeconomic status, which could be related to exposure to advertisements and the capability to buy infant formula (18). In the MICS 2003, a greater proportion of illiterate women was exclusively breastfeeding (37%) compared to women who had some education (32.4%) which was marginally significant (p=0.053). Tribal women were also more likely to be exclusively breastfeeding (53.6%).

Delayed initiation of breastfeeding was common in Bangladesh but rates of initiation have improved in recent years. In a study published in 1996, only 9% of women had initiated breastfeeding immediately after birth (16). In a study of rural women conducted in 1996, 27% of women initiated breastfeeding on the third day or later (18). In the MICS 2003, 88.8% of the mothers had initiated breastfeeding within the first three days. The recent DHS survey confirms these results with 24% of women initiating breastfeeding within one hour of birth and 83% of women initiating breastfeeding one day after delivery (15). These encouraging results with respect to initiation of breastfeeding suggest that health-promotion messages provided to birth attendants and pregnant women on the importance of early initiation of breastfeeding are working.

The prevalence of prelacteal feeding remains high in Bangladesh. In a study of 2,105 mothers in rural Bangladesh, 85% had fed their child prelacteal food, and only 10% fed colostrum during the child's first three days of life (18). In a study published in 1992, less than 8% of mothers gave colostrum as a first food to their child. The majority of mothers who did not feed colostrum believed that it caused diarrhoea or stomach upsets (19). Although feeding colostrum is reported to be uncommon in other studies, in this survey, it was likely that prelacteal foods, such as honey or sweetened water, were given with colostrum. Giving sweet prelacteals is thought to be related to the belief that these will ensure a pleasant personality (20). In the MICS 2003, 66.6% of the women fed their children prelacteal feeds of honey, sugar water, or mustard oil, and yet a high proportion (88.8%) fed their child breastmilk within the first three days showing that many children were fed colostrum.

There are many limitations to this study. The most appropriate use of cross-sectional data is to collect information about the burden of illness in a community. Since the outcome (morbidity) and the study factor (exclusive breastfeeding) are collected at the same time, causation cannot be inferred, and any measured associations may be susceptible to bias. Confounding occurs when factors relating to breastfeeding practices are also related to morbidity (21). Common confounding variables include socioeconomic status, maternal age, or education status, and presence of clean water and sanitation facilities. We adjusted estimates for a number of variables that were available given the cross-sectional nature of the data. However, we were not able to correct for maternal age other factors that may have been associated with morbidity, for example maternal nutrition or low birth-weight. When interpreting results from cross-sectional datasets, the problem of reverse causality may also be a source of bias. For example, if mothers tended to breastfeed exclusively because the child was ill, the effect of exclusive breastfeeding on illness would have been underestimated. Conversely, if mothers stopped breastfeeding as a result of illness, this would have biased the results towards an overestimation of an effect. Other sources of bias include interviewer- and responder-bias and the seasonality of the survey and the fact that only infants who survived up to the time of the interview were included in the analysis. These factors could not be corrected for in these analyses.

Apart from these limitations, the advantages of cross-sectional datasets, such as those provided by MICS and DHS are the frequency with which they are collected (usually every 2-3 years), the standardized nature of questionnaires, and the representative characteristics of sample. The datasets are ideal for assessing trends but can also be used for determining associations if confounding factors are controlled for. In addition, wide dissemination of the MICS results could be used as an opportunity for encouraging governments to re-assess their priorities in regard to children's well-being, to promote breastfeeding, and to achieve the goals of the Global Strategy on Infant and Young Child Feeding and the Millennium Development Goals.

In summary, the MICS 2003 data showed that, among surviving children, exclusive breastfeeding was significantly associated with decreased morbidity from diarrhoea and acute respiratory infection in Bangladesh. Rates of early initiation of breastfeeding are improving. However, the giving of prelacteal foods, such as honey or sugared water, is still a popular cultural tradition in Bangladesh, and this reduces the true prevalence of exclusive breastfeeding. The MICS 2003 data showed that only 11.7% of the infants aged less than three months were exclusively breastfed according to the WHO definition which is far below the recommended level. In a country where almost 70% of infant deaths are attributable to acute respiratory infection or diarrhoea (1), improving the prevalence of exclusive breastfeeding has the potential to substantially decrease child morbidity and mortality and is imperative to improve rates of child survival.
